# Motor Imagery-Related Changes of Neural Oscillation in Unilateral Lower Limb Amputation

**DOI:** 10.3389/fnins.2022.799995

**Published:** 2022-05-19

**Authors:** Xinying Shan, Jialu Li, Lingjing Zeng, Haiteng Wang, Tianyi Yang, Yongcong Shao, Mengsun Yu

**Affiliations:** ^1^School of Biological Science and Medical Engineering, Beihang University, Beijing, China; ^2^Beijing Key Laboratory of Rehabilitation Technical Aids for Old-Age Disability, National Research Center for Rehabilitation Technical Aids, Beijing, China; ^3^School of Psychology, University of Leeds, Leeds, United Kingdom; ^4^School of Psychology, Beijing Sport University, Beijing, China

**Keywords:** lower limb amputation, EEG, brain remodeling, motor imagery, neural oscillations, spectral analysis

## Abstract

An amputation is known to seriously affect patient quality of life. This study aimed to investigate changes in neural activity in amputees during the postoperative period using neural electrophysiological techniques. In total, 14 patients with left lower limb amputation and 18 healthy participants were included in our study. All participants were required to perform motor imagery paradigm tasks while electroencephalogram (EEG) data were recorded. Data analysis results indicated that the beta frequency band showed significantly decreased oscillatory activity in motor imaging-related brain regions such as the frontal lobe and the precentral and postcentral gyri in amputees. Furthermore, the functional independent component analysis (fICA) value of neural oscillation negatively correlated with the C4 electrode power value of the motor imagery task in amputees (*p* < 0.05). Therefore, changes in neural oscillations and beta frequency band in motor imagery regions may be related to brain remodeling in amputees.

## Introduction

With the rise in natural disasters, motor vehicle accidents, and malignant diseases, an increased number of patients with traumatic limb injuries have undergone amputation surgery. There will be phantom limb pain after amputation, which is known to seriously affect patient quality of life. Compared with non-amputees, most amputees have poor physical and mental health and encounter more obstacles in their daily lives (World Health Organization, 2011). Amputation is an irreversible process. Limb loss leads to changes in sensory input to the central nervous system, resulting in a series of maladjustment challenges such as limitations in self-care ability, and frequent mental health issues (Waight et al., 2015).

Partial severance of the peripheral nervous system due to amputation results in remodeling of the entire nervous system (Williams et al., 2016). In the early postoperative period, many amputees experience phantom limb sensations (Kelle et al., 2017), and a series of dynamic changes in brain neural activity subsequently occurs (Molina-Rueda et al., 2013). For example, one resting-state electroencephalogram (EEG) study showed that changes in the neural network of patients with right-sided amputations weakened the inhibitory effect of the sensorimotor area, and that connectivity of the right parietal lobe area increased (Lyu et al., 2016). Moreover, recent studies have shown that amputees have structural and functional changes to multiple brain areas, such as the premotor cortex, and the supplementary motor area (Molina-Rueda et al., 2019). These structural and functional changes can be interpreted as non-adaptive brain remodeling due to sensory deprivation and loss or decreased autonomous control ability (MacIver et al., 2008; Raffin et al., 2016). Therefore, a study of brain remodeling after amputation is likely to facilitate understanding of the neural mechanisms of amputation-related abnormal sensation and may help to guide patient rehabilitation through promoting benign brain remodeling, as well as provide a theoretical basis for the development of new prostheses, thereby improving patient quality of life.

Among available non-invasive electrophysiological techniques, multi-channel EEG is widely used. However, to understand brain plasticity in amputees and provide more precise results, advanced technologies are needed in the future to identify the mechanisms involved in sensorimotor cortex neural activity following amputation. Functional independent component analysis (fICA), which has been widely used to compare the changes in resting state networks among individuals in functional magnetic resonance imaging (fMRI) technology (Lv et al., 2018), provides an opportunity to investigate the mechanisms involved in post-amputation neural activity. In addition to fMRI technology that can only indirectly measure neural activity, EEG has also been used to study neural oscillations in resting-state networks (Liu et al., 2017; Samogin et al., 2019). Ye et al. (2019) indicated that patients with somatoform pain disorder showed greater resting alpha oscillations in the parietal areas. However, few studies have used resting-state EEG to explore neural oscillations in amputees. By analyzing neural activity in the resting-state, it is possible to investigate changes in the neural oscillations of the sensorimotor area of amputees at different frequencies; this can be beneficial in providing neurophysiological indicators for future rehabilitation and intervention of amputees. Therefore, fICA method allows for a potentially more accurate determination of the effect of amputation on brain plasticity from an electrophysiological perspective.

Brain remodeling is considered to be related to abnormal motor imagery after amputation (Anderson and Lenz, 2011). One potential cause of impaired motor imagery is the change in body schema due to the phantom limb. Event-related spectral perturbation is often used to describe the neural characteristics (e.g., brain oscillation) of motor imagery tasks (Cebolla et al., 2015). In amputees with phantom limb perception, event-related desynchronization of EEG in the beta band (beta-ERD) in central and parietal areas showed angular disparity (i.e., the beta-ERD weakened monotonically with orientation from 0° to 180°) (Lyu et al., 2016), which suggested that phantom limb perception during the task might be an important confounder for motor imagery in amputees. Additionally, motor imagery technology has been widely used in many areas, including in the treatment of phantom limb pain (PLP) (Moseley, 2006), motor rehabilitation (Priganc and Stralka, 2011), and the development of the brain–computer interface (BCI) technology for the control of prostheses (Thomas et al., 2013). Moreover, when performing lower limb movement/motor imagery tasks, many researchers mainly focused on C3, Cz, and C4 electrodes (Pfurtscheller et al., 2013; Yi et al., 2013). Therefore, our study combined EEG with the motor imagery paradigm to analyze the dynamic changes in the cerebral cortex following an amputation, and explore the effect of PLP on this change. This provided a preliminary theoretical basis for the development of non-invasive BCI, based on EEG and the popularization of prosthesis in the future.

This study is the first to analyze the changes of neural oscillation and EEG power in patients with lower limb amputation by combining resting state and motor imagery induced EEG, so as to reveal brain plasticity and its effect on motor imagery function after amputation. We aimed to investigate the changes of neural oscillation in resting state network in patients with lower limb amputation by using fICA method, and to study the effect of PLP on EEG power during motor imagery to provide a neurophysiological and psychological basis for the study of BCI based on EEG. Based on previous studies, we hypothesized that amputation would lead to remodeling of sensorimotor regions, especially in terms of decrease in the contralateral frontal lobe and central gyrus. Moreover, the motor imagery ability of patients would decrease after amputation, and this may be related to PLP.

## Materials and Methods

### Participants

Fourteen participants with left lower limb amputations (mean age, 51.93 ± 11.54 years; females, *n* = 2) were selected through the Affiliated Hospital of the National Research Center for Rehabilitation Technical Aids and 18 healthy participants were also recruited (mean age, 44.89 ± 10.34 years; females, *n* = 3). All participants grew with a common geographic background (Beijing, China) and had the same ethnic identification (the Han Chinese), while they were people from different socioeconomic and education backgrounds. Independent sample *t*-test results indicated no significant difference in age between the two groups (*p* = 0.09). The average number of months having lived with an amputation was 11.64 ± 7.59 months in the amputation group, and PLP grades of the 14 amputees were calculated using a visual analog scale (VAS) with a six-point score (from 0 to 5; average PLP grade, 1.79 ± 1.57) (Table 1). All the amputations had been due to accidental trauma, none of the participants had any motor sensory dysfunction or other pathophysiological diseases, and there was no history of any neurological or psychiatric disorders. This study was approved by the Ethics Committee of the National Research Center for Rehabilitation Technical Aids, and the participants provided written informed consent prior to participation in the study.

**TABLE 1:** Detailed clinical characteristics of amputees.

Participants	Gender	Age	Level of amputation	Side	Time since amputation (month)	Phantom limb pain (level)
1	Male	66	Transtibial	Left	16	3
2	Male	58	Transtibial	Left	16	0
3	Male	49	Transtibial	Left	16	2
4	Female	57	Transtibial	Left	20	0
5	Male	51	Transtibial	Left	30	3
6	Female	70	Transfemoral	Left	5	0
7	Male	63	Transfemoral	Left	15	2
8	Male	57	Transfemoral	Left	6	1
9	Male	54	Transfemoral	Left	15	4
10	Male	51	Transfemoral	Left	7	3
11	Male	45	Transfemoral	Left	3	5
12	Male	29	Transtibial	Left	6	1
13	Male	48	Transtibial	Left	4	0
14	Male	29	Transfemoral	Left	4	1
Mean		51.93			11.64	1.79
*SD*		11.54			7.59	1.57

### Resting-State and Task-State Electroencephalogram

Resting- and task-state EEG data acquisition was performed for all 32 participants (14 amputees) in this study. The setup of EEG headset took 18 min, including soaking in KCl Solution for 15 min and wearing the headset for 3 min. After being comfortably seated, participants were asked to remain quiet for 2 min, and to relax. They were then asked to maintain an open eye state, during which EEG data acquisition was undertaken for 5 min.

After resting-state EEG acquisition was completed, task-state EEG data acquisition was undertaken based on a foot motor imagery paradigm, which is an experimental paradigm based on left lower limb motor imagery and designed using E-Prime 2.0. There were 32 trials in our study (the duration was 8 min), and each trial with a “+” in the center of the screen for the first 2 s was used to draw the participant’s attention and then prompted the appearance of an icon to the left, at which point the participant engaged in 10 s of left lower limb motor imagery, that is, imagining up and down movement of the toe on the left foot. Subsequently, there was a 3-s resting-state.

### Electroencephalogram Recording

High-density EEG was recorded using an electrode net (Geodesic Sensor Net, Electrical Geodesics Inc., Eugene, OR, United States) comprising 256 electrodes interconnected with thin rubber bands, and containing small sponges soaked with saline water, which were in direct contact with each participant’s scalp surface.

**FIGURE 1:**
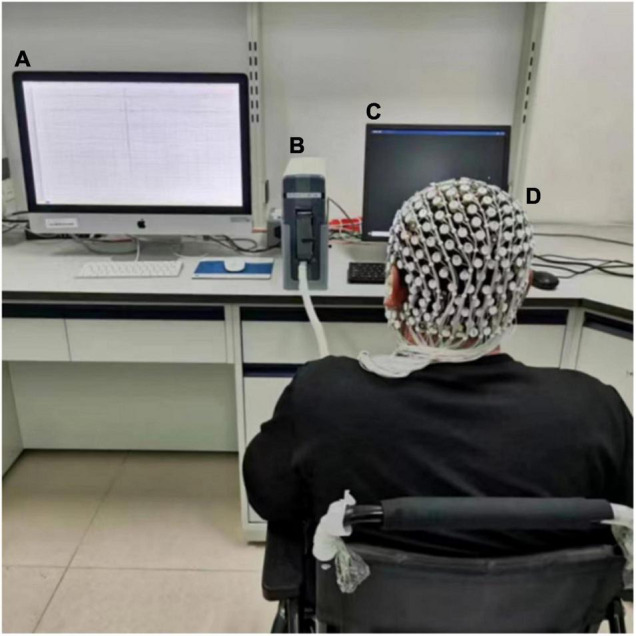
Experimental setup. **(A)** Data acquisition; during EEG recording, this device maintained a black screen. **(B)** EEG Amplifier. **(C)** Monitor. **(D)** 256-electrode cap.

EEG data were acquired with a 256-channel HydroCel Geodesic Sensor Net (EGI, Eugene, OR) using Net Station 4.5 software (Figure 1). All electrode impedances were below 50 kΩ before the recording was started, and the band filters were applied between 0.1 and 30 Hz during continuous recording, which included a 50 Hz notch filter to remove power supply noise. EEG recordings were sampled at 1,000 Hz.

### Data Analysis

**FIGURE 2:**
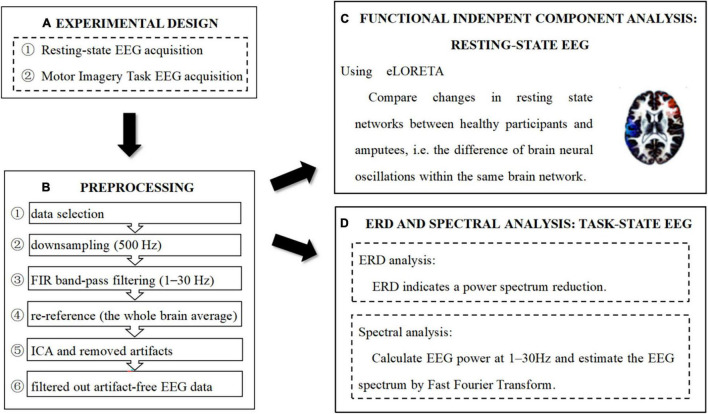
Experimental design, preprocessing, and analysis pipeline. **(A)** Experimental design. **(B)** Preprocessing pipeline. **(C)** Functional independent component analysis: resting-state EEG. **(D)** ERD and Spectral analysis: task-state EEC.

The summary of the entire procedure of data processing is presented in Figure 2.

#### Electroencephalogram Data Preprocessing

Preprocessing of EEG data was performed using the toolbox EEGLAB with MATLAB 2015b software, as follows: (i) nineteen electrods (FP1/2, F3/4, F7/8, Fz, C3/4, Cz, T3/4, T5/6, P3/4, Pz, O1/2) of the international 10–20 system were selected, (ii) the sampling rate was downsampled to 500 Hz, (iii) filter impulse response (FIR) band-pass filtering was performed and set to 1–30 Hz (transition bandwidth = 1 Hz; order = 7,420), (iv) the whole brain average reference was used for re-reference, (v) data were analyzed using independent component analysis (ICA) to remove components that presented significant artifacts, (vi) ADJUST1.1.1 plug-in within EEGLAB was used to semi-automatically remove independent components with obvious artifacts, and (vii) data containing obvious drift or artifacts were removed manually, and less than 10% of each data segment was removed. Artifact-free EEG data were then filtered out.

#### Functional Independent Component Analysis: Resting-State Electroencephalogram

Functional independent component analysis using exact low resolution brain electromagnetic tomography (eLORETA) was undertaken to compare changes in brain neural oscillations in healthy participants and amputees at rest, within the same brain network (Glahn et al., 2005; Aoki et al., 2015). During fICA, each distinct pair of scalp electrodes was analyzed and the “similarity” between the signals was computed. If two signals were very similar, i.e., highly correlated-coherent-synchronized, they were assigned to the same network. Here, the results of the cortical electrical distributions of networks were rather different from those of known functional networks (Chen et al., 2013). Finally, all distinct pairs of intracranial signals were scanned. The Montreal Neurological Institute (MNI) brain volume was scanned at 5 mm resolution by eLORETA software. This produced 6,239 cortical gray matter voxels at 5 mm resolution. The technical details can be found in Aoki et al. (2015).

Briefly, we calculated the electrocortical activity of the brain network in terms of the following frequency bands: delta, 1.5–6 Hz; theta, 6.5–8 Hz; alpha-1, 8.5–10 Hz; alpha-2, 10.5–12 Hz; beta-1, 12.5–18 Hz; beta-2, 18.5–21 Hz; and beta-3, 21.5–30 Hz (Pascual-Marqui, 2002; Katayama et al., 2018). With this initial procedure, each participant contributed seven eLORETA images of cortical spectral density (one for each frequency band). In addition, the brain network automatically divided into 7 networks (i.e., 7 components of interest), and each participant could generate 7 coefficients (one coefficient for each network, expressing how that participant uses that network). Each network image would display areas whose activities are tightly linked (i.e., they work together as a network). From the point of view of mathematics, these data correspond to a “function” of space (cortical voxel) and frequency. In the next step, the data from each participant were concatenated, thus producing a matrix where one dimension corresponds to different participants and the other jointly corresponds to space-frequency. Neural activity was then calculated as the global field power value (Pascual-Marqui, 2002) under each condition (network).

Subsequently, statistical analysis was performed on these seven coefficients, comparing the amputation and healthy groups, in search of how the amputees’ brain used the resting state networks differently from the healthy brain. Subsequently, an independent sample *t*-test was used to compare the coefficients of the healthy and amputation groups. During the *t*-test, the randomization Statistical non-Parametric Mapping (SnPM) was performed, which was suitable for multiple comparisons, and no other correction method was needed; meanwhile, the corrected critical thresholds and *p*-values were computed automatically (Painold et al., 2020). Finally, the brain networks with significant differences were found, and the coefficient of the significant network was derived, which was called fICA value (i.e., the global field power value with significant difference between the amputation and healthy groups).

#### Event-Related Desynchronization Analysis and Spectral Analysis: Task-State Electroencephalogram

Time–frequency representations of power were computed using wavelet analysis. This was performed by convolving the complex Morlet wavelets with the single-trial EEG data (Pfurtscheller et al., 2006). We used wavelets with frequencies ranging from 1 to 30 Hz in steps of 1 Hz. In this study, the C4 and Cz electrodes were associated with left lower limb movement; we extracted the average value of the alpha frequency band (7–12 Hz) and the low beta frequency band (13–20 Hz) on the C4 and Cz electrodes to plot the waveform. Power was calculated as the squared norm of the resulted frequency-specific time courses of complex numbers. Power was averaged across trials and then normalized as change relative to the mean power in a baseline interval from −1 to 0 s, using the following method. Relatively long epochs (−2 to 5 s) were used for time-frequency decomposition to allow edge artifacts to subside outside the time window of interest (−1 to 4 s); thereafter, data outside this time window were trimmed off. The detailed calculation method of ERD can be found in Graimann and Pfurtscheller (2006).

Spectral analysis of the preprocessed task-state EEG data was performed using the toolbox STEP 1 [Single Trial detection toolbox for Epilepsy (Version 1.0)] (Hu et al., 2011) with MATLAB 2015b software. First, the epoch time range was set to 0–10 s, and the zero of an epoch is the start of motor imagery, and the frequency range was set to 1–30 Hz. Then, we selected the channel C3, Cz, and C4, respectively, and calculated EEG power values at 1–30 Hz [four frequency bands: delta (1–4 Hz), theta (4–8 Hz), alpha (8–13 Hz), and beta (13–30 Hz)]. The EEG spectrum is estimated using Fast Fourier Transform.

#### Statistical Analyses

SPSS 25.0 software was used to perform the independent sample *t*-test on the power values of the healthy group and the amputation group in delta (1–4 Hz), theta (4–8 Hz), alpha (8–13 Hz) and beta (13–30 Hz) frequency band under the C3, Cz, and C4 electrodes, respectively. Subsequently, the correlations of the fICA values and power values with significant differences in the two groups were compared using Pearson’s correlation analysis.

Pearson’s correlation analysis was also used to explore the relationship between the level of PLP and the power value of amputees in delta (1–4 Hz), theta (4–8 Hz), alpha (8–13 Hz), and beta (13–30 Hz) frequency band under nineteen electrods of the international 10–20 system.

## Results

### Neural Oscillation During Resting-State Electroencephalogram

**FIGURE 3:**
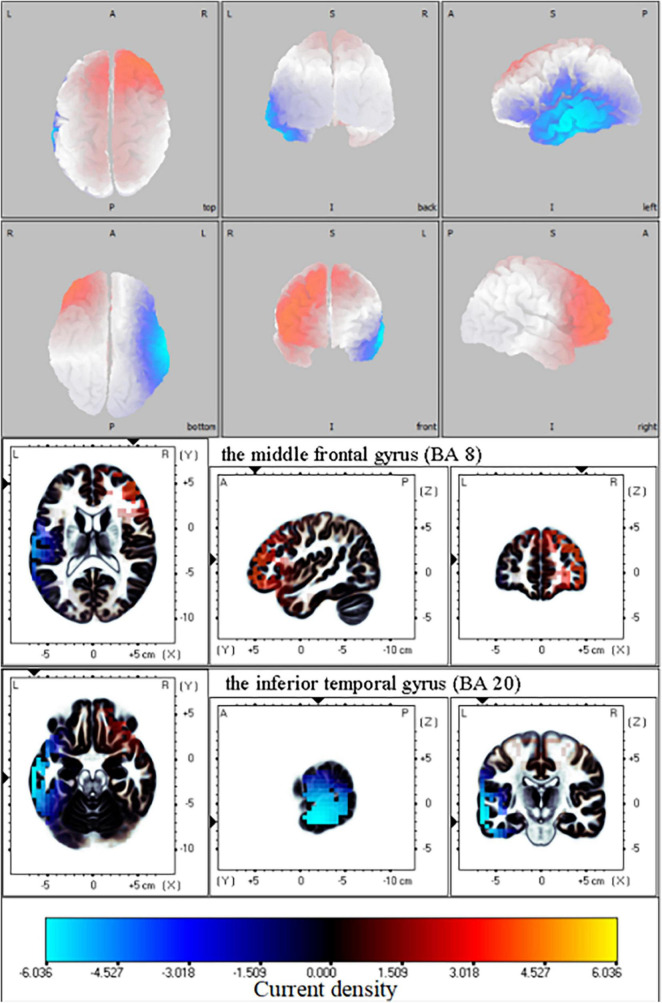
Functional independent component analysis (fICA): Neural oscillation changes in resting state. Changes in right frontal lobe and left temporal lobe information transmission between amputation group and healthy group in beta-3 band (21.5—30 Hz); The bright yellow indicates increased beta oscillation in amputee group, while blue color denotes the beta oscillation was weakened in amputee group relative with healthy group. L, left; A, anterior; R, right; P, posterior; S, superior; I, inferior.

Only one network difference between the amputation group and healthy group was found in this study [threshold (*t*) = 1.70, *p* = 0.02] (Figure 3). In this network, no significant alterations in neural activity were detected in the delta, theta, or alpha frequency segments according to the fICA results. The results showed that contralateral frontal beta-3 oscillations were significantly enhanced in the amputation group compared with the healthy group, especially in the middle and superior frontal gyri [Brodmann area (BA) 8, *t* = 3.55]. Furthermore, we also observed significantly reduced beta oscillations in the ipsilateral frontal, temporal, and parietal lobes of the amputees, with the most significant difference observed in the inferior temporal gyrus (BA20, *t* = −6.18). In addition, brain regions showing reduced beta-3 oscillations in amputees included the postcentral (BA43, *t* = −4.66), precentral (BA6, *t* = −4.48), middle occipital (BA37, *t* = −3.86), and fusiform gyri (BA37, *t* = −4.47). Since only one significant difference network was found in our study, the fICA value of each participant was the coefficient of the network.

### Electroencephalogram Analysis During Motor Imagery

**FIGURE 4:**
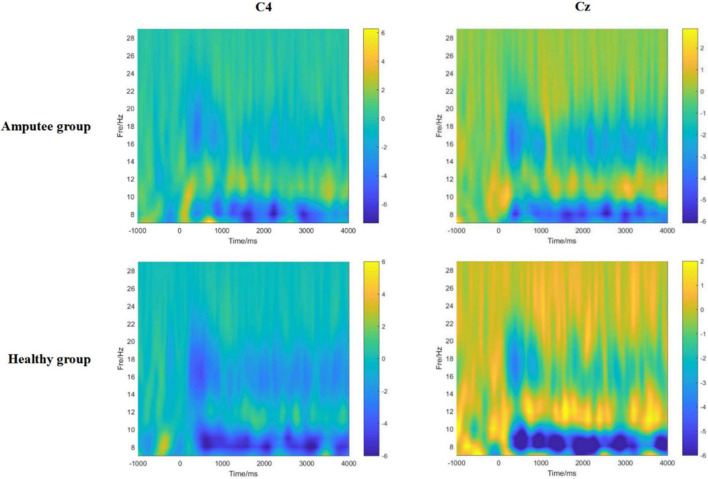
The time-frequency diagrams of the primary motor cortex (C4, Cz) of amputees and healthy controls during the motor imagery task. A significant mu rhythm (7–12 Hz) and low beta frequency (13–20 Hz) after stimulation can be observed in both amputees and healthy controls.

Through the time-frequency analysis of motor imagery in patients with left lower limb amputation and healthy controls (Figure 4), it was found that at the Cz electrode and contralateral C4 electrode, neural oscillation activity significantly decreased in the alpha frequency band and low beta frequency band (ERD). Therefore, motor imagery of healthy people and amputees was both successfully evoked in this study.

The results of spectral analysis showed the average and the standard deviation of power values at each frequency segment between amputees and healthy participants in this study (Table 2). We compared the difference in power values between amputee and healthy participants at C3, Cz, and C4 electrodes when performing motion imagery in four frequency bands, namely, delta, theta, alpha, and beta. After a Bonferroni correction for multiple comparisons, *p* would need to be < 0.017 to be significant. The independent sample *t*-test results showed significant differences in power values at Cz (*t* = 2.519, *p* = 0.017) in the beta frequency band, and the difference at C4 (*t* = 2.130, *p* = 0.042) in the beta frequency band is significant at edge. Therefore, the follow-up analysis of this study only focuses on Cz-beta and C4-beta power values.

**TABLE 2:** Difference in power values (mean ± SD) and the changes in power values of task-state EEG between healthy group and amputation group.

Frequency band	Electrode	Amputation group	Healthy group	T	*p*
Delta	C3 Cz	0.14 ± 0.57 0.18 ± 0.10	0.16 ± 0.11 0.22 ± 0.25	−0.873 −0.550	0.389 0.586
	C4	0.14 ± 0.06	0.16 ± 0.10	−0.572	0.571
Theta	C3 Cz	0.08 ± 0.02 0.11 ± 0.05	0.08 ± 0.04 0.10 ± 0.06	−0.153 0.277	0.879 0.784
	C4	0.07 ± 0.02	0.07 ± 0.05	−0.327	0.746
Alpha	C3 Cz	0.10 ± 0.04 0.13 ± 0.08	0.08 ± 0.05 0.11 ± 0.07	1.086 0.550	0.286 0.587
	C4	0.08 ± 0.03	0.08 ± 0.04	0.577	0.568
Beta	C3 Cz	0.06 ± 0.07 0.06 ± 0.02	0.04 ± 0.02 0.04 ± 0.01	1.320 2.519	0.197 0.017*
	C4	0.06 ± 0.04	0.04 ± 0.01	2.130	0.042*

Delta (1–4 Hz), theta (4–8 Hz), alpha (8–13 Hz), and beta (13–30 Hz); *p < 0.05.

### Correlation Between Changes in Functional Independent Component Analysis Value and Changes in Power Value

**FIGURE 5:**
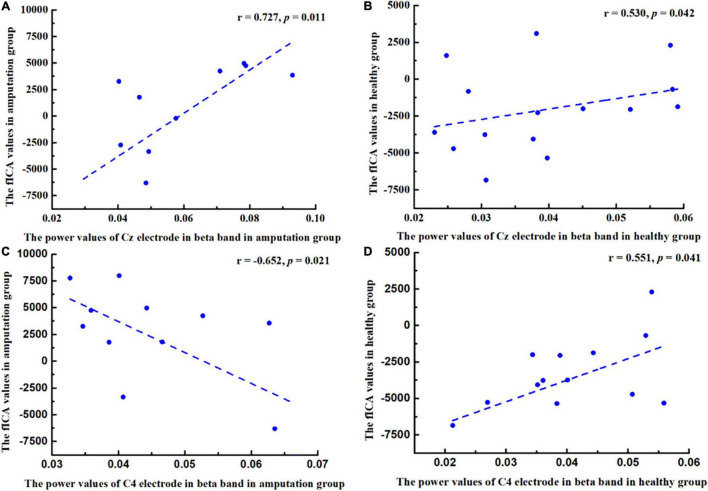
Pearson correlation coefficient between changes in power values of beta band (13–30 Hz) and changes in functional independent component analysis (fICA) values. **(A)** A positive correlation was found between Cz power values and fICA values in amputee group (*r* = 0.727, *p* = 0.011). **(B)** A positive correlation was found between Cz power values and fICA values in healthy group (*r* = 0.530, *p* = 0.042). **(C)** A negative correlation was found between C4 power values and fICA values in amputee group (*r* = −0.652, *p* = 0.021). **(D)** A positive correlation was found between C4 power values and fICA values in healthy group (*r* = 0.551, *p* = 0.041).

This study calculated the relationship between fICA values and power values (Cz-beta and C4-beta) in the amputation and healthy groups separately using Pearson’s correlation analysis, and found that in the amputation group, there was a significant positive correlation between fICA value and Cz-beta power value (*r* = 0.727, *p* = 0.011), while the fICA value negatively correlated with C4-beta power value (*r* = −0.652, *p* = 0.021) (Figures 5A,C). In the healthy group, the fICA value positively correlated with the Cz-beta power value (*r* = 0.530, *p* = 0.042) and the C4-beta power value (*r* = 0.551, *p* = 0.041) (Figures 5B,D).

### Correlation Between Changes in Power Value and Phantom Limb Pain Level

**FIGURE 6:**
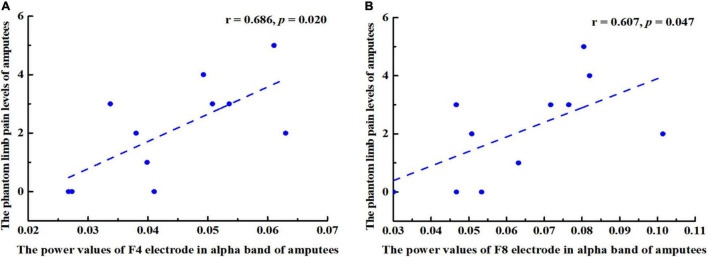
Pearson correlation coefficient between changes in power values of alpha band (8–13 Hz) and the phantom limb pain (PLP) levels. **(A)** A positive correlation was found between F4 power values and PLP levels in amputees (*r* = 0.686, *p* = 0.020. **(B)** F8 power values and PLP levels were positively correlated (*r* = 0.607, *p* = 0.047).

In this study, the PLP level of amputees was positively correlated with the power value of right frontal cortex in the alpha band (Figure 6), especially at F4 (*r* = 0.686, *p* = 0.020) and F8 (*r* = 0.607, *p* = 0.047).

## Discussion

This study aimed to investigate the changes in neural activity and function in brain regions related to motor imagery in left lower limb amputees from the perspective of resting state network and functional density changes through combining resting-state and task-state EEG. The results showed that at the Cz and C4 electrode, which is related to motor function, there was significant difference in the power of beta frequency between amputation group and healthy group, whereas the neural oscillation activity of brain areas related to motor imagery such as the frontal lobe, and the anterior and posterior central gyri, were significantly decreased. In addition, in the amputation group, the fICA value of neural oscillation positively correlated with the Cz electrode power value in relation to the motor imagery task, while, the opposite results were observed at C4 electrode. Previous studies have shown a series of adaptive changes in the brain post-amputation, which may be related to functional remodeling of the brain.

In this study, resting-state EEG data were collected with participants’ eyes open to compare changes in cerebral neural oscillation activity between amputees and healthy participants. In contrast with the healthy participants, we observed that the contralateral frontal lobe beta neural oscillation activity of amputees was significantly enhanced, especially in the middle frontal and superior frontal gyri. However, neural oscillation activity in the ipsilateral occipitotemporal region, related to visual processing, and the central anterior posterior gyrus and supplementary motor area, related to motor imagery, had weakened. Oscillatory activity in the beta frequency band is considered a unique function of attention and sensorimotor control (Chung et al., 2016). After amputation, to achieve effective control of the stump, regions of an amputee’s brain undergo dynamic changes simultaneously. These regions involve the area of visual sensory input, the sensory area, and the motor area of sensory output. Furthermore, we found that participants’ brain activity in the temporal lobe changed significantly, which may have been be due to the use of a prosthesis in daily life or due to the process of rehabilitation, resulting in the remodeling of visual sensorimotor areas related to prosthesis use. For example, van den Heiligenberg et al. (2018) found that the greater the use of the prosthesis in daily life, the stronger the activity of the visual motor sensory selection area in the lateral occipitotemporal cortex, which indicated that the change in daily motor behavior had contributed to the shape formation of the motor areas related to prosthesis use. However, due to the lack of actual movement in an amputee’s residual limb, individual motion feedback becomes increasingly more incomplete, which increasingly promotes the generation of individual illusions, leading to more significant functional remodeling of the brain. Our major finding was that, in the beta frequency band, there was a weakening of neural oscillations in the precentral and postcentral gyri. We presume that this change may be due to the joint effect of amputation on the sensory input and output pathways. However, it is not clear how the sensorimotor area and the neural oscillation of the visual network coordinate to process visual and sensorimotor information and control movement after brain remodeling.

We investigated the EEG spectral analysis density of amputees during motor imagery of the amputated limb to show energy changes in electrodes related to motor imagery (C3, Cz, and C4) after amputation. We found that there was a significant difference between amputees and healthy participants at the power value of the Cz/C4 electrode in the beta frequency range (13–30 Hz). Our results showed that although amputees could not perform the same exercises as the healthy participants, they still retained the ability to imagine the residual limb. Moreover, the reuse of motor imagery has been shown to promote motor recovery and reduce PLP (Saruco et al., 2019). However, after amputation, motor imagery is regulated through phantom limb sensations (Lyu et al., 2016), which reflects the adaptation process of the brain post-amputation. MRI studies have shown that in patients with lower limb amputation, hemispheric pathways that contribute to motor coordination and imagination are reorganized, especially in the anterior motor area and the supplementary motor area (Li et al., 2017). Motor imagery is the same as real movement and can play a rehabilitation role in the motor ability of amputees. To regain control of the residual limb and ensure the high-frequency use of healthy limbs, the neural activity of the cerebral cortex changes adaptively, which is mainly reflected in remodeling of the sensorimotor area. However, in the early stages of amputation, brain remodeling can lead to decreased motor control or inhibition. When motion planning and execution occurs following an amputation, traditional contralateral motor area activation is reconstructed and occurs in the posterior parietal lobe area, which may reflect the adaptation of visual spatial feedback to motion control after amputation (Lyu et al., 2016). Our results indicate that beta energy enhancement of the Cz/C4 electrode is an important factor affecting motor imagery after amputation, which we consider is related to adaptive remodeling of the brain.

Pearson’s correlation analysis was used to explore the relationship between the fICA and power values in the healthy and amputation groups. We found a significant correlation between the two in the amputation group at Cz and C4 electrode. These results indicated that the activity of neural oscillations in the relevant brain regions of amputees was closely related to the energy changes triggered through motor imagery tasks. Brookes et al. (2011) have found that neural oscillations mediate neural activity in functional networks. EEG spectral analysis can reflect the functional status of each brain region, and changes in power values represent energy changes in this brain region. In amputees, the activity of neural oscillations in the frontal lobe, precentral gyrus, and postcentral gyrus decreased significantly, and the beta power value of the central area increased during the motor imagery task. We consider that the neural activity of the functional network of brain regions related to motor imagery in amputees was impaired, which is consistent with other studies reporting that brain structure and function in amputees were impaired in areas of the brain related to motor imagery, such as the premotor and supplementary motor areas (Molina-Rueda et al., 2019). In the motor imagery task, the beta power of the amputees was significantly enhanced in the central area of the brain. Therefore, the amputees improved the energy of the beta band in the motor imagery task to compensate for the deficiency of the functional network of the sensorimotor area of the brain due to amputation, which reflects the compensatory function of the brain when the amputees imagine movement of the left lower limb. In addition, Jiang et al. (2015) have found that the connectivity of the contralateral sensorimotor cortex in amputees decreased significantly. However, in this study, decreased beta neural oscillations were observed in the ipsilateral motor imagery related areas of the amputees, which also indicated that the ipsilateral related areas of the brain were reorganized to compensate for the motor imagery of the amputees.

This study had some limitations. First, the number of participants was small, and we only studied participants with left lower limb amputation; therefore, our study findings cannot be representative of all amputees. Second, a small number of trials in the motor imagery paradigm were performed in this study. Third, since there were many noisy channels among the 256 channels, only 19 electrodes were selected for fICA analysis in our study. In addition, we focused only on the C3, Cz, and C4 electrodes for the spectral analysis. However, the use of more electrodes may provide more useful information to explore the changes in sensorimotor networks. It will be beneficial to investigate the effects of PLP and motor imagery ability on neural activity of the sensorimotor brain regions for different limbs in future studies based on a larger sample size in combination with fMRI technology; thereafter, the number of trials in the paradigm should be increased to improve the accuracy of subsequent experiments.

## Conclusion

In this study, resting-state EEG data were collected in relation to a motor imagery task, and the correlation between neural oscillation and brain energy was explored using fICA and spectral analysis. In the amputee group, neural oscillations in the ipsilateral sensorimotor area were associated with functional reorganization in the cerebral cortex, and a decrease in neural oscillation negatively correlated with a change in energy in beta frequency during motor imagery. Consequently, there were adaptive changes in neural activity in the cerebral cortex in each amputee, along with certain compensatory activities, especially a decrease of neural oscillations in the beta frequency of the precentral and postcentral gyri.

## Data Availability Statement

The raw data supporting the conclusions of this article will be made available by the authors, without undue reservation.

## Ethics Statement

The studies involving human participants were reviewed and approved by the National Research Center for Rehabilitation Technical Aids. The patients/participants provided their written informed consent to participate in this study.

## Author Contributions

MY and YS designed the study. XS and JL produced the results and wrote the manuscript. LZ, HW, and TY contributed data collection and analysis. YS, XS, and MY were the guarantors of this study. All authors listed have read and approved the final manuscript.

## Conflict of Interest

The authors declare that the research was conducted in the absence of any commercial or financial relationships that could be construed as a potential conflict of interest.

## Publisher’s Note

All claims expressed in this article are solely those of the authors and do not necessarily represent those of their affiliated organizations, or those of the publisher, the editors and the reviewers. Any product that may be evaluated in this article, or claim that may be made by its manufacturer, is not guaranteed or endorsed by the publisher.
